# Deduction of Back Pain Patients Using EMG Technology and Inertial Sensors During Functional Tests

**DOI:** 10.3390/s26061882

**Published:** 2026-03-17

**Authors:** Philipp Floessel, Freya Charlotte Wunderlich, Jil-Justin Funke, Hannes Kaplick, Jan Jens Koltermann, Alexander C. Disch

**Affiliations:** 1 University Center for Orthopedics, Trauma & Plastic Surgery—Section Sports Medicine and Rehabilitation, Faculty of Medicine Carl Gustav Carus, Technische Universität Dresden, Fetscherstrasse 74, 01307 Dresden, Germany; 2 University Center for Orthopedics, Trauma & Plastic Surgery—University Comprehensive Spine Center (UCSC), Faculty of Medicine Carl Gustav Carus, Technische Universität Dresden, Fetscherstrasse 74, 01307 Dresden, Germany; charlywunder@yahoo.de (F.C.W.); jil-justin.funke@ukdd.de (J.-J.F.); jan.koltermann@mailbox.tu-dresden.de (J.J.K.); alexander.disch@ukdd.de (A.C.D.)

**Keywords:** functional assessment, low back pain, wearable inertial sensors, electromyography sensors

## Abstract

Low back pain (LBP) represents an immense economic burden, with a lifetime prevalence of up to 84%. However, conventional diagnostic methods such as Magnetic Resonance Imaging (MRI) or X-rays provide only limited information about the pathogenesis and specific pain-related functional limitations. Wearable inertial sensors (IMU) and electromyography sensors (EMG) offer an expanded spectrum for the targeted identification and diagnosis of LBP. The aim of the study is to develop and evaluate a standardized multi-sensor functional assessment protocol for the subcategorization of functional deficits in LBP. Based on a systematic literature review, a standardized and objectively measurable functional LBP assessment protocol was defined that tests fatigue resistance, neuromuscular control, lumbopelvic stability, and global trunk musculature. Subsequently, 38 individuals were recruited in a prospective cross-sectional study and divided into three groups: “healthy,” “mild pain,” and “severe pain.” These individuals underwent an assessment. The two pain groups differed significantly from the symptom-free individuals in all previously defined functional levels. In addition, the two pain groups also differed significantly from each other. The functional assessment, which incorporates IMUs and EMG sensors as central diagnostic elements, enables the identification of functional deficits and associated neuromuscular characteristics, thus enabling individualized therapy.

## 1. Introduction

In the most developed economies, approximately 75% of jobs and value creation are attributable to the service sector [1]. This sector is dominated by sedentary office work [2], which promotes physical inactivity and, in the medium to long term, general physical deconditioning [3]. Consequently, in service-based societies, back pain is among the most common causes of work incapacity and represents a considerable health burden for affected individuals [4]. Lifetime prevalence is reported to be as high as 84% [5]. In the age cohort between 35 and 50 years, initial degenerative pain and inflammatory processes of the spine frequently occur [6]. This age group is therefore particularly prone to acute back complaints [7]. Owing to the substantial economic impact, pain-related absenteeism in these individuals leads to significant productivity losses and considerable socioeconomic costs [4].

To prevent the chronification of pain, and to derive and evaluate targeted therapeutic exercises, precise determination of functional deficits is essential. Conventional clinical diagnostic methods, such as MRI or radiography, provide only limited information on the pathogenesis of back pain and on specific pain-associated functional restrictions [8]. Visible structural changes of the spine often result in misinterpretation as the primary pain source, even though they are not causative for the symptoms. Patients who undergo MRI in the early phase of pain are more likely to catastrophise their symptoms, which in turn increases the risk of chronicity [9]. In the vast majority of patients (approximately 90–95%), back pain is triggered by muscular tension, postural dysfunction, or psychosocial factors (stress, burden) and cannot be visualized by imaging [10,11]. Nevertheless, recent systematic reviews indicate that imaging has increased over the past 20 years and that at least one third of all imaging procedures are unnecessary [12,13].

Beyond the clinical assessment of musculoskeletal disorders, diagnosing the functional status of pain patients under load—as well as quantifying and classifying functional deficits based on objectively measurable parameters—remains challenging [14].

Descriptions of functional limitation frequently rely on patient-reported, questionnaire-based assessments such as the Oswestry Disability Index and the Roland–Morris Disability Questionnaire [15,16]. A universally accepted diagnostic set-up that measures the functional status of trunk-stabilizing musculature under standardized and thus reproducible loading conditions, while providing objective outcome parameters, is lacking [10]. However, recent systematic reviews highlight the relevance of the anatomy and function of the trunk-surrounding musculature—and, in particular, the quality of neuromuscular innervation of the deep autochthonous multifidus muscles—in the development and chronification of back pain [17,18].

At both functional and structural levels, the acute onset and the chronification of low back pain (LBP) can be characterized by distinct changes [19]. Compared with healthy individuals, patients with LBP may exhibit reduced fatigue resistance [20], diminished neuromuscular control [21], altered lumbopelvic stability [22], and lower strength capacity of the trunk-stabilizing musculature [23]. Nevertheless, functional deficits need not encompass all four domains but may occur at varying severity and in different combinations, potentially reinforcing one another [24]. Functional tests that mirror everyday loading demands are therefore particularly suitable for specifying these deficits [25].

The continued development of digital technologies—such as inertial measurement units (IMUs), wearable electromyography (EMG), and portable force plates—enables objective, real-time acquisition of biomechanical data during movement. Among others, Shakourisalim et al. and O’Sullivan et al. demonstrated that such objective data possess predictive validity for back pain risk [26,27].

The technological suitability of IMUs for capturing complex biomechanical loads on the spine is supported by current validation studies. For example, Shakourisalim et al., in their study on estimating muscle forces in the lower back, were able to demonstrate that IMU-based systems are capable of mapping muscle forces during functional lifting tasks with an accuracy that correlates closely with the established gold standard of optical motion capture [27].

Furthermore, [28] demonstrate in their validation study that the use of IMU sensors for the objective differentiation between individuals with and without chronic low back pain (CLBP) based on lumbopelvic kinematic parameters is possible. Through the analysis of 141 subjects, the study shows that mobile wearables can identify significant differences in movement speed and acceleration (AUC) and thus serve as a predictive tool for clinical classification [27].

Furthermore, the review by [29] underscores the paradigm shift from isolated laboratory measurements to instrumented functional diagnostics using wearable technologies in the diagnosis of low back pain. The synergistic combination of electromyography (EMG), inertial measurement units (IMUs), and force plates for capturing the “motor signature” overcomes the limitations of previous standard clinical tests. The study demonstrates that this technological triangulation enables a profound objectification of pain mechanisms under realistic conditions [29].

This is particularly relevant given that in over 80% of patients, back pain is defined as “non-specific.” This illustrates that a purely structural view of the spine is insufficient for diagnosis and clinical decision-making. Instead, functional deficits, which manifest in specific phenotypes, are coming into focus. Clinically, these are characterized by impairments in maximum trunk strength, reduced muscular fatigue resistance, deficits in neuromuscular control, and impaired lumbopelvic stability [24,30].

Although numerous studies since 2015 have demonstrated the relevance of these individual functional dimensions, a validated “ready-to-go” assessment that simultaneously captures all these subcategories in a cohort of participants is still lacking in routine clinical practice. While technological advancements in inertial measurement units (IMUs) and surface electromyography (sEMG) provide the hardware basis for such multidimensional diagnostics, a standardized, evidence-based protocol that integrates both systems into a coherent phenotyping tool is still lacking [27,29,31,32].

Therefore, the aim of this study is to develop and evaluate a standardized multi-sensor functional assessment protocol integrating IMU and EMG measurements for the phenotyping of low back pain. The proposed approach combines clinically established functional tests with wearable sensor technology to enable differentiation between healthy individuals and pain subgroups and to identify specific functional deficits relevant for clinical decision-making. It is hypothesized that this integrated assessment provides a more comprehensive and coherent functional profile than the isolated application of individual diagnostic methods.

## 2. Materials and Methods

### 2.1. Participants

A total of 38 participants (65.8% female) were included in the study. The mean age was 43 years (SD ± 11), mean height was 174 cm (SD ± 8), and mean weight was 76 kg (SD ± 15). Among these, 29 participants reported nonspecific chronic low back pain, as assessed using the Chronic Pain Grade Questionnaire (CPGQ, see Section 2.2) developed by Korff [33]. Based on the CPGQ pain intensity scores, participants were stratified into three subgroups: 0 to 10, 11 to 29, and >29. The general characteristics of the sample are presented in Table 1.

To accommodate both German- and English-speaking participants, the CPGQ was administered in the appropriate language version

Participants were excluded if they were unable to maintain a single-leg stance for at least 60 s, had significant musculoskeletal impairments (e.g., joint replacements, spinal deformities), had severe neurophysiological disorders (e.g., epilepsy), and reported acute pain intensity > 8 on a Visual Analogue Scale (VAS).

Recruitment was carried out using flyers and posting in medical offices between October 2024 and January 2025. Only datasets that included complete execution of all tasks and full sensor recordings were included in the analysis. One participant was excluded due to technical failure during data collection.

### 2.2. Study Design

**Figure 1:**
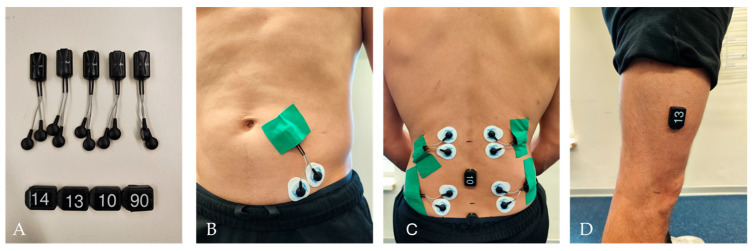
Sensor placement and preparation for EMG and IMU measurements. (**A**) Five EMG sensors (upper part) and four IMU sensors (lower part). (**B**) Placement of the EMG sensor for recording activity from the anterior superior iliac spine (ASIS). (**C**) Two EMG sensors on the right and left strands of the longissimus thoracis (LT) (upper part), two EMG sensors on the right and left strands of the lumbar multifidus (MF) (lower part), two IMU sensors at the level of L3 and S1 (middle and lower part). (**D**) IMU thigh approx 15 cm above the knee joint space (placed on the right and left leg).

This prospective cross-sectional observational study was conducted to evaluate a standardized multi-sensor functional assessment protocol for the characterization of functional deficits in individuals with and without low back pain. The primary objective was to examine whether the integrated assessment protocol allows differentiation between healthy individuals and pain subgroups across multiple functional domains. Each participant attended a single-day session in a German University Hospital. Prior to data collection, participants underwent a standardized medical screening by a trained physician to ensure eligibility. The Functional Assessment Screening Protocol (Table 2) was based on previous publications of this group and systematic literature research conducted. The complete search strings are provided in Appendix A. The measurement system required for the assessment consists of five EMG sensors (Figure 1A) and four IMU sensors (Figure 1A).

Before EMG electrode placement, the skin was prepared by shaving (if needed), scrubbing and disinfecting with 70% alcohol. EMG data were collected bilaterally from the lumbar multifidus (Figure 1C) and longissimus thoracis (Figure 1C) muscles. To minimize signal crosstalk, electrodes were positioned well within muscle borders, aligned parallel to muscle fibers, set with a 2 cm inter-electrode distance. The skin impedance was accepted if less than 55 kΩ. Electrode placement followed SENIAM guidelines and validated procedures [34]. The lumbar multifidus (MF) electrodes were aligned along the anatomical line from the posterior superior iliac spine to the L1 and L2 interspace at the L5 spinous process level. The longissimus thoracis (LT) electrodes were placed two finger widths lateral to the L1 spinous process. A reference electrode was placed over the right anterior superior iliac spine (ASIS) (Figure 1B) [34,35].

Four triaxial inertial measurement units (IMU) were affixed to the following anatomical landmarks: Trunk at L3 and S1 levels (Figure 1C), and thigh approx 15 cm above the knee joint space (Figure 1D). All sensors’ attachments were performed by certified physiotherapists and sports scientists.

Participants performed the assessments in a standardized sequence to ensure uniform measurement conditions for all subjects. The individual tests are presented in chronological order in Table 2.

### 2.3. Experimental Procedures and Measures

At the start of each session, participants were briefed on the study objectives. A medical examination was conducted, including vital signs (heart rate, blood pressure), demographics, physical activity in the past 12 h, and whether the assessment had an endoprothesis, followed by a measurement of the body composition using a bioelectrical impedance analysis (TANITA). Prior to the functional assessments, participants had to fill out the CPGQ to evaluate the pain intensity over the past three months [33].

Outcomes were derived from synchronized IMU, EMG sensors and force plate-based Center of Pressure (CoP) data. Each task was explained to the participants in detail prior to execution. Participants completed a trial run and were required to demonstrate their understanding before official data collection began.

#### 2.3.1. Fatigue Resistance/Strength Endurance—Gait Analysis

Strength endurance was assessed using a 100 m walking test. The test subjects were asked to cover the distance at a brisk pace of their choice. The participants wore their usual outdoor shoes. During all walking phases, gait parameters were recorded using IMUs and EMG. The outcome variables included: walking speed, cadence, and gait cycle time, as well as the duration of the stance and swing phase [36].

#### 2.3.2. Neuromuscular Control and Range of Motion (ROM)

**Figure 2:**
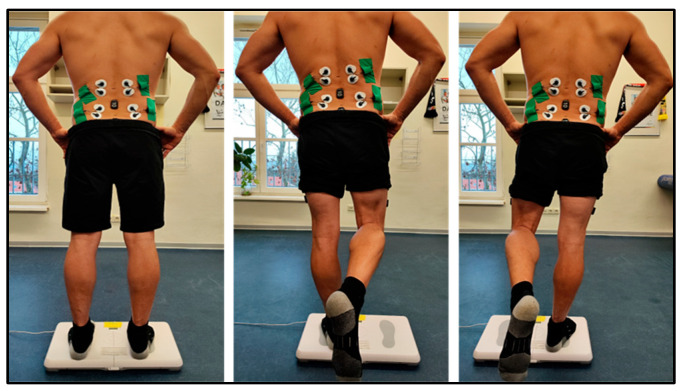
Assessment of postural control for the evaluation of neuromuscular control during double-leg and single-leg stance.

The neuromuscular control was assessed using postural control measurements during quiet bipedal stance, single-leg stance (left and right in randomized order), and quiet sitting on a force plate. Each trial lasted 60 s, with a 10 s rest period between trials. All measurements were conducted barefoot with participants’ hands on hips and eyes open. A visual target was placed 3 m away at a height of 1.7 m. Foot placement was standardized using visual markers, and during single-leg stance, the non-supporting leg was not allowed to touch the stance leg. Participants were instructed to maintain an upright and steady trunk posture throughout the trial. The primary outcomes were CoP-track, Power Density Analyze (PSD) within the frequency range of 0.02–10.0 Hz, and IMU and EMG parameters recorded from the MF and LT muscles (Figure 2).

**Figure 3:**
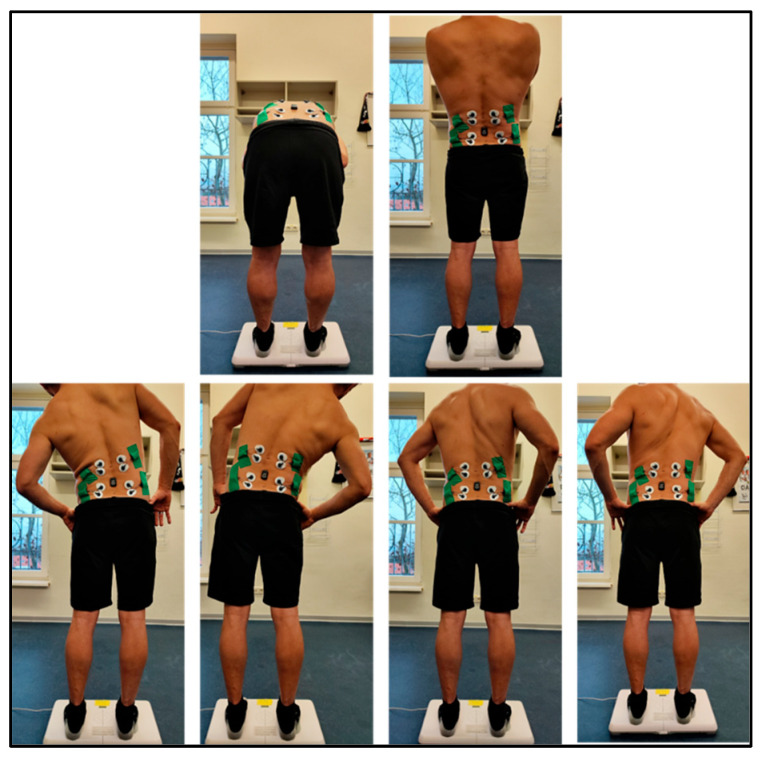
Assessment of functional spinal movements in stance.

**Figure 4:**
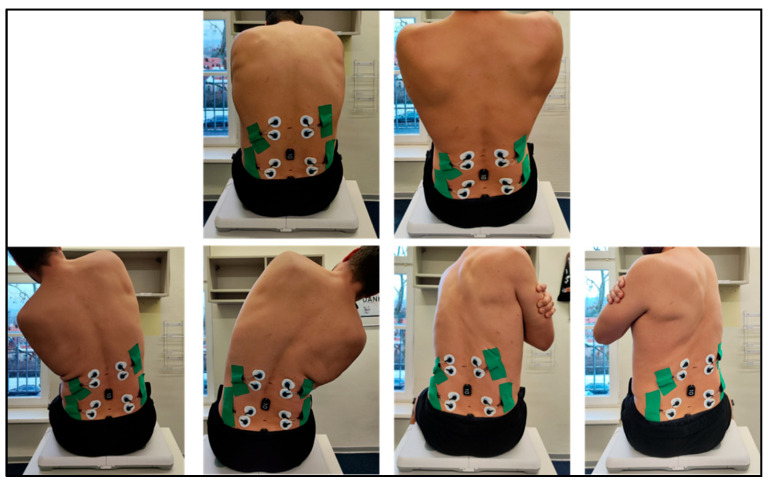
Assessment of functional spinal movements in a seated position on a balance board (lateral flexion, rotation, and flexion/extension).

All functional spinal movements were assessed in a fixed sequence: flexion, extension, lateral flexion (left/right, self-selected order), and rotation (left/right, self-selected order). Each motion was performed twice, with a 3 s pause in the neutral spinal position between trials. Testing was conducted in both standing and sitting postures (Figure 3 and Figure 4). Adjacent joint movements were restricted through verbal guidance. Trials lasted as long as needed, followed by short rest periods. Movements were recorded using IMU and EMG. Participants were barefoot, with hands crossed over the shoulders and knees extended during standing tests.

#### 2.3.3. Lumbopelvic Stability

**Figure 5:**
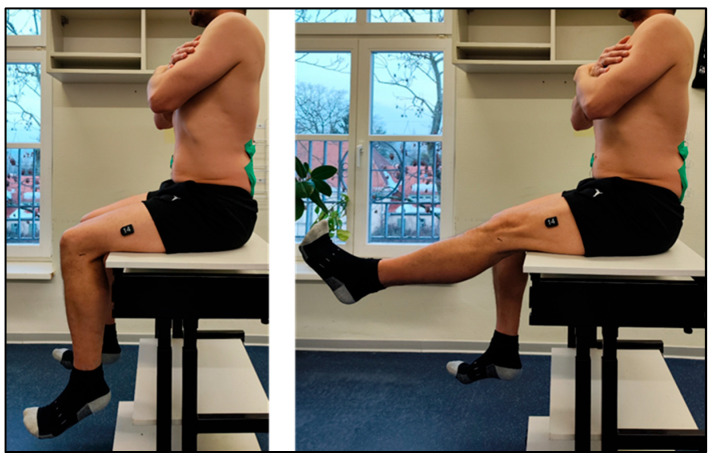
Assessment of functional lumbopelvic stability in seated position.

Lumbopelvic stability was assessed using a modified slump test. Participants sat upright (defined as anterior rotation of the pelvis, lumbar lordosis, and relaxation of the thorax, hands crossed on the shoulders), with thigh fully supported. Participants then extended each leg five times with dorsiflexed ankles, alternating sides. Subjective outcome parameters were the ability to maintain upright posture and the presence of pain or neurological symptoms. Additionally, the objective outcomes that were collected were IMU and EMG parameters at the MF and LT muscles (Figure 5).

#### 2.3.4. Global Trunk Musculature

Isometric trunk strength assessments were conducted using an Isomed 2000 dynamometer. Participants were seated in an upright position with the hip angle fixed at 90°. The thighs and lower legs were stabilized using device-specific fixation systems to minimize compensatory movements and isolate trunk muscle activity. Prior to the isometric strength trials, participants completed a warm-up phase involving 20 repetitions of dynamic trunk flexion and extension under isokinetic conditions at 60°/s and ROM 40° (10–30°).

**Figure 6:**
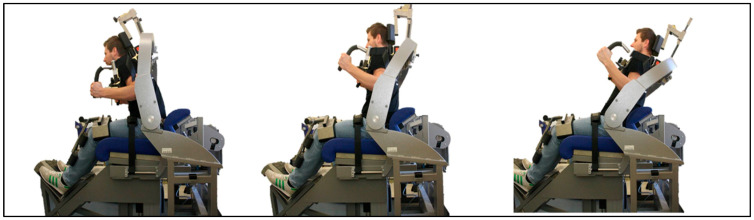
Assessment of isometric trunk strength in seated position.

For the isometric strength assessment, five standardized joint angle positions were tested. For ventral trunk muscles, two angle positions were tested: 1. Neutral starting position; 2. 80° hip flexion. For the dorsal trunk muscles, three angle positions were tested: 1. Neutral starting position; 2. 100° hip flexion; 3. 115° hip (Figure 6).

At each angle, participants were instructed to exert maximal voluntary contraction while maintaining the prescribed posture. Outcome parameters included the peak torque (Nm), the average torque (Nm), and the total force output (Nm). All measurements were recorded and stored for further statistical analysis.

### 2.4. Data Acquisition and Statistical Analysis

#### 2.4.1. EMG and IMU System “Cometa”

Surface EMG (sEMG) was used to capture MF and LT muscle activity during gait, CoP tasks and the slump test. Before EMG recording, each participant was instructed to follow the imminent task. The electrodes were connected to an EMG data collection system with the wireless apparatus of a Cometa miniwave infinity device (Cometa slr, Milan, Italy), and the signals were collected using customized software named EMG and Motion Tools, Inc. software version 7 (Cometa slr, Milan, Italy). Data analysis and processing were performed using the raw EMG signa

Data collection was performed using a custom acquisition setup integrated with MATLAB 2024 (MathWorks Inc., Natick, MA, USA). A detailed description of all extracted IMU, EMG, and CoP features is provided in Appendix B. Raw sensor signals from inertial measurement units (IMUs), electromyography (EMG), and force platforms were imported into MATLAB for preprocessing and parameter extraction. Signal conditioning included filtering, segmentation, and normalization according to established biomechanical standards. Derived parameters—such as kinematic slopes, variability indices, and EMG envelopes—were computed using custom scripts and validated routines.

#### 2.4.2. CoP Measuring System

CoP data were recorded using modified Wii Balance Boards (Prototype, Potsdam, Germany) (sampling frequency 1 kHz; a 14-bit-resolution) [37], which have been validated for CoP measurements [38]. The primary outcome was CoP track length (in cm). Outliers were defined as values exceeding 1 × 10^7^ or deviating more than 90 Raw data were recorded and structurally stored using custom software developed in LabVIEW 2022. The CoP trajectory was computed following signal preprocessing, including applications of a third-order low-pass Butterworth filter [39]. The cutoff frequency is calculated according to Koltermann et al. [40].

#### 2.4.3. Statistics

Beyond classical group comparisons, the study had a pronounced exploratory component, owing to the exceptionally high temporal resolution and the large number of data points recorded synchronously from IMU and EMG across all functional assessments (gait, postural control/CoP, and flexion–extension bending). Each participant contributed 1872 gait data points, 1782 CoP data points, and 2106 bending-related samples, yielding a highly granular, multimodal dataset that enabled a detailed, multidimensional assessment of neuromuscular control and movement dynamics within the same temporal window. This density of within-subject, time-aligned measurements enhances the ability to identify subtle patterns, temporal irregularities, and domain-specific movement signatures that may not be detectable through summary metrics alone.

However, the richness of the dataset contrasts with the relatively small overall sample size (*n* = 38), which limits statistical power at the between-subject level. Consequently, emphasis was placed not only on significance testing via one-way ANOVA, but also on effect size estimation (e.g., partial eta-squared) and on the consistency of findings across the large number of within-subject observations. While the high sampling density supports robust exploratory interpretations, inferential conclusions must nevertheless be drawn cautiously—particularly given the multiple outcome domains analyzed (gait, postural control, bending, lumbopelvic stability, and isometric strength), which may increase the risk of inflated type-I error.

Due to the exploratory nature of the study, no formal multiplicity correction (e.g., Holm–Bonferroni or FDR) was applied; however, results were screened for robustness across parameter families to mitigate the risk of false positives. All statistical analyzes were conducted using R statistical software version 4.1.0 (R Foundation for Statistical Computing, Vienna, Austria).

To account for inter-individual differences in body size and mass, all isokinetic trunk strength values were normalized to body weight and expressed as Newton meters per kilogram (Nm·kg^−1^). This approach enables a more accurate comparison of trunk flexion and extension capacity between participants and is widely recommended in biomechanical and clinical research to ensure that torque values reflect true neuromuscular performance rather than variations in absolute body mass. Normalization therefore improves comparability across pain groups and supports more valid interpretation of strength imbalances.

Importantly, all recorded variables were statistically tested, but in accordance with common reporting standards for exploratory biomechanics and neuromuscular research, only those outcomes that demonstrated statistically significant between-group differences are presented in the manuscript. Non-significant findings were reviewed for plausibility and internal consistency but were omitted from reporting to maintain clarity and focus on the most robust indicators of functional impairment.

### 2.5. Ethic

The research project was approved by the Ethics Committee of Dresden University of Technology (approval number BO-EK-215052022_1) and conducted in accordance with the Declaration of Helsinki. All participants provided their written consent prior to participation after receiving detailed information about the study procedure and data protection guidelines.

## 3. Results

### 3.1. Fatigue Resistance/Strength Endurance—Gait Analysis

Significant differences were observed in both acceleration metrics of the lower extremity and pelvis, as well as in EMG parameters of the MF (Table 3).

For the accelerometer data, marked increases in dynamic measures were evident with rising pain intensity. In particular, the maximum slope of acceleration at the left thigh (max_slopeR) was approximately 180% higher under high-intensity pain compared with the pain-free condition (*p* = 0.012; power = 0.759). Similarly, variability along the *Z*-axis at the left thigh (varL) increased significantly (*p* = 0.042), as did the maximum slope at the pelvic axis (Pelvis_Acc_X; *p* = 0.043).

Conversely, EMG parameters of the MF demonstrated a reduction in activity with higher pain intensity. The mean EMG value on the left (meanR) decreased from 0.0009 (pain-free) to 0.0005 (high-intensity pain; *p* = 0.050), while the area under the curve on the right (aucR) also declined (*p* = 0.046).

The reported post hoc power values, ranging from approximately 0.49 to 0.76, indicate a moderate sensitivity of the statistical tests to detect true between-group differences. Parameters with higher power (e.g., max_slopeR with power = 0.759) suggest that the observed effects are likely robust and less prone to type-II error. In contrast, variables with power values around 0.50 (e.g., MF meanR or aucR) should be interpreted more cautiously, as the probability of missing true effects is higher in these cases. Importantly, the moderate power reflects the relatively small sample size of the study but does not diminish the consistency of effect directions across related metrics. The convergence of findings across multiple IMU and EMG parameters strengthens confidence in the overall pattern, even when individual tests exhibit only moderate power.

### 3.2. Neuromuscular Control

During the single-leg stance, increasing pain intensity was associated with greater ranges of fluctuation and a spectral shift toward higher frequencies (Table 4). The spatial fluctuation values increased significantly: the fluctuation area increased by ~175% and the fluctuation ellipse by ~132% from the pain-free to the painful state (*p* = 0.033 and *p* = 0.024; power = 0.599 and 0.654, respectively). Spectral energy increased in both the low- and high-frequency bands (FreqBandEnergy_L: +57%, *p* = 0.023, power = 0.661; FreqBandEnergy_H: +87%, *p* = 0.019, power = 0.691), with a simultaneous increase in the mean frequency along the *X*-axis (+66%; *p* = 0.048, power = 0.505) and the dominant frequency along the *Y*-axis (≈+300%; *p* = 0.049, power = 0.497). Power along the *Y*-axis increased (+34%; *p* = 0.031, power = 0.608), while power along the *X*-axis decreased (−52%; *p* = 0.027, power = 0.634). Despite the increased range of fluctuation, the total fluctuation distance and speed decreased (−23% and −58%; *p* = 0.050 and *p* = 0.040; power = 0.523 and 0.570).

The statistical power associated with the single-leg stance variables ranged from approximately 0.50 to 0.69, indicating a moderate ability to detect true group differences. Parameters with higher power—such as the spectral energy measures (FreqBandEnergy_L and FreqBandEnergy_H, power ≈ 0.66–0.69)—provide relatively strong evidence for genuine differences in neuromuscular control patterns. In contrast, variables with power values closer to 0.50 (e.g., MeanFreq_X or DominantFreq_Y) should be interpreted with greater caution due to a higher probability of type-II error. Importantly, the convergence of significant effects across multiple independent domains (spatial sway, spectral energy, directional power, and frequency components) supports the robustness of the observed pattern, even when individual power levels are only moderate. This consistency strengthens the overall interpretation that increasing pain intensity is associated with a shift toward higher-frequency, stiffer, and more effortful postural control.

During single-leg stance, pain intensity was associated with significant changes in the minimum EMG envelope and raw signal values, reflecting altered tonic activation patterns (Table 5). The right internal oblique muscle showed a significant increase in its minimum envelope (minENV) from −7.49 in the pain-free state to −0.05 in the low-pain state and 0.55 in the high-pain state (*p* = 0.033, power = 0.601). Similarly, the right multifidus showed a progressive increase in minENV (−2.07 → −0.46 → 1.60; *p* = 0.012, power = 0.755). In contrast, the left multifidus showed the opposite trend, with its minimum envelope decreasing from 2.12 to −0.47 (*p* = 0.001, power = 0.934), suggesting asymmetric modulation of tonic control.

The power values associated with these EMG parameters ranged from 0.47 to 0.93, indicating varying sensitivity to detect true group differences. The left multifidus (minENV), with a power of 0.934, represents a highly reliable and well-supported effect, suggesting a strong and consistent alteration of tonic control with increasing pain. The right multifidus and right internal oblique also demonstrate moderate to high power (0.755 and 0.601), supporting the robustness of the observed shifts in baseline activation. In contrast, the thoracic erector spinae parameters exhibited lower power (≈0.47–0.53), indicating that although their directional trends were consistent with compensatory recruitment, these findings should be interpreted cautiously due to a higher likelihood of type-II error. Importantly, the convergence of significant effects—particularly in the multifidus bilaterally—strengthens the conclusion that pain is associated with a systematic reorganization of tonic trunk muscle activation during single-leg stance.

### 3.3. Mobility/Bending

In a seated forward bending task (results Table 6) with an additional accelerometry at the lumbar spine, increasing pain intensity was associated with systematic reductions in lumbar and pelvic kinematics and acceleration-derived metrics, alongside higher minimal MF activation. At the lumbar spine, peak and range of X-axis acceleration decreased from 1.185 to 0.84 (−29%) and from 1.18 to 0.85 (−28%), respectively (LWS_ACC_X_MAX: *p* = 0.024, power = 0.658; LWS_ACC_X_RANGE: *p* = 0.030, power = 0.623). Lumbar roll kinematics were likewise reduced (LWS_ROLL_MAX: 78.44 to 60.99, −22%, *p* = 0.019, power = 0.690; LWS_ROLL_RANGE: 81.93 to 58.26, −29%, *p* = 0.023, power = 0.665), and the integrated lumbar acceleration (LWS_ACC_X_AREA) declined from 3.02 to 1.92 (−36%; *p* = 0.037, power = 0.582). Pelvic dynamics showed parallel changes, with decreases in X-axis acceleration peak and range (PELVIS_ACC_X_MAX: 0.940 to 0.64, −32%, *p* = 0.030, power = 0.620; PELVIS_ACC_X_RANGE: 0.93 to 0.64, −31%, *p* = 0.030, power = 0.623) and in pelvic roll maximum (58.19 to 42.95, −26%; *p* = 0.028, power = 0.632). The strongest statistical signal was observed for the minimum pelvis pitch (LWS_PELVIS_PITCH_MIN), which fell markedly from 4.47 to 0.06 (≈−99%; *p* = 0.003, power = 0.905), consistent with a substantial constraint in sagittal plane excursion. In contrast to the kinematic attenuation, the minimum EMG envelope of the MF muscles increased with pain on both sides (right: 3.14 to 6.66, +112%, *p* = 0.049, power = 0.512; left: 2.79 to 7.99, +186%, *p* = 0.034, power = 0.598).

Across the seated bending metrics, statistical power ranged from 0.51 to 0.91, indicating moderate to high sensitivity to detect true group differences. Notably, the strongest effect was observed for pelvis pitch minimum (power = 0.905), reflecting an exceptionally robust impairment of sagittal plane control in pain groups. Other kinematic parameters (power ≈ 0.62–0.69) demonstrate moderately strong evidence, suggesting that reductions in lumbar and pelvic acceleration and roll are reliable findings despite the modest sample size. In contrast, EMG-derived variables showed only moderate power (≈0.51–0.60), indicating that while the direction of effects is consistent and physiologically plausible, these specific estimates should be interpreted with some caution due to a higher potential for type-II error. Crucially, the coherent pattern across multiple independent metrics—reduced kinematic excursion combined with increased minimal MF activation—strengthens confidence in the interpretation that pain is associated with a more constrained, protective bending strategy.

During the return from forward flexion to an upright posture (Table 7), pain intensity was associated with pronounced reductions in lumbar and pelvic acceleration profiles and angular excursions, alongside increased thoracic and paraspinal muscle activation. Lumbar acceleration area (LWS_ACC_X_AREA) declined substantially from 4.03 in the pain-free condition to 2.37 under high pain (−41%; *p* = 0.012, power = 0.753), with a similar trend in a second measure (3.90 to 2.24; *p* = 0.005, power = 0.845). The minimum pelvis pitch (LWS_PELVIS_PITCH_MIN) exhibited the strongest attenuation, falling from 6.50 to 0.09 (≈−99%; *p* = 0.009, power = 0.798), while integrated pitch and roll ranges were also reduced (LWS_PELVIS_PITCH_AREA: 216.87 to 125.03, −42%, *p* = 0.021; LWS_PITCH_AREA: 426.20 to 286.77, −33%, *p* = 0.031; LWS_ROLL_RANGE: 102.96 to 62.29, −39%, *p* = 0.031). Pelvic acceleration and roll areas mirrored these reductions (PELVIS_ACC_X_AREA: 2.91 to 1.68, −42%, *p* = 0.017; PELVIS_THIGH_ROLL_AREA: 175.35 to 117.24, −33%, *p* = 0.018).

Conversely, EMG metrics indicated heightened muscular engagement with pain. The mean and RMS envelopes of the thoracic ES increased progressively (R_THORACICES_ENV_MEAN: 13.46 to 23.08, +72%, *p* = 0.035; R_THORACICES_ENV_RMS: 15.51 to 26.43, +70%, *p* = 0.039), and the duty cycle of the left MF rose from 0.71 to 0.88 (+24%; *p* = 0.042). Minimal EMG envelope values for the right MF more than doubled (2.33 to 6.02, +158%; *p* = 0.016), and raw thoracic EMG also increased (*p* = 0.036).

The power values across the extension-phase bending metrics ranged from 0.56 to 0.85, indicating moderate to high sensitivity to detect true group differences. Effects with higher power—particularly the reductions in lumbar acceleration (power = 0.753–0.845) and pelvis pitch (power = 0.798)—can be considered robust and unlikely to represent type-II errors. Mid-range power values (≈0.60–0.71), observed for most pelvic and thoracic kinematic parameters, still support meaningful effects but warrant somewhat more cautious interpretation. Lower-to-moderate power levels for several EMG variables (≈0.56–0.59) indicate that while their directionality is physiologically consistent with compensatory co-contraction, the statistical certainty of these effects is comparatively weaker. Importantly, the convergence of kinematic and EMG findings—restricted sagittal motion combined with increased tonic extensor activation—reinforces the interpretation that individuals with pain adopt a protective, stiffened extension strategy, even where individual tests exhibit only moderate power.

### 3.4. Lumbopelvic Stability

During the seated straight-leg-raise test (SSLR) (see Table 8), the left multifidus showed a significant reduction in the minimum envelope (minENV) across groups (1.812 → 0.435 → −0.830 a.u.; *p* = 0.033, power = 0.60), indicating a progressive shift towards lower minima with increasing pain. This pattern is compatible with impaired tonic support and delayed/insufficient low-level activation of the multifidus during hip flexion, aligning with a weakened lumbopelvic control contribution by the deep extensors. 

By contrast, right internal oblique and left thoracic erector spinae exhibited progressive increases in mean raw EMG with pain (R_Int_Oblique meanRAW: −1.76 × 10^−4^ → 4.86 × 10^−4^ → 7.21 × 10^−4^; *p* = 0.051, power = 0.51; L_ThoracicEs meanRAW: −4.92 × 10^−4^ → 3.94 × 10^−5^ → 7.43 × 10^−4^; *p* = 0.052, power = 0.536). Although these trends are borderline and do not meet conventional significance (α = 0.05), they are directionally consistent with compensatory recruitment of superficial trunk muscles during SSLR when MF muscles support is diminished.

Taken together, the SSLR EMG pattern suggests a pain-related reorganization of trunk muscle control characterized by reduced minimal activation of the (left) multifidus and increased mean activation of abdominal/thoracic extensors. This is consistent with a shift in lumbopelvic stabilization from deep to more superficial musculature during the task, which may reflect a compensatory but less efficient control strategy.

The power values for the SSLR EMG parameters ranged from 0.41 to 0.60, indicating low-to-moderate sensitivity to detect true effects. The significant left-multifidus finding (power = 0.60) has acceptable robustness despite the small sample size. In contrast, the right internal oblique and left thoracic ES showed notably lower power (~0.41–0.51), suggesting a higher likelihood of type-II error and that these effects, while physiologically plausible, should be interpreted cautiously. The consistent directional pattern across muscles, however, supports the broader interpretation of a compensatory shift from deep to superficial stabilizers during SSLR, even where individual statistical power is limited.

### 3.5. Core Strength

Peak flexion torque at 0° was higher in the high pain group compared with healthy controls (1.67 vs. 1.28 Nm·kg^−1^; +31%; *p* = 0.033, power = 0.607), while the comparison at 10° did not reach significance despite a numerically similar trend (1.58 vs. 1.24 Nm·kg^−1^; *p* = 0.702, power = 0.067). In contrast, peak extension torque was reduced in pain groups at 0° and 15° (0°: 2.62 vs. 3.01 Nm·kg^−1^, −13%; *p* = 0.029, power = 0.634; 15°: 2.73 vs. 3.19 Nm·kg^−1^, −14%; *p* = 0.016, power = 0.724), whereas the difference at 30° was not significant (2.82 vs. 3.13 Nm·kg^−1^; *p* = 0.879, power = 0.053) (Table 9).

Across the isometric strength assessments, statistical power ranged widely (0.053–0.724), indicating variable sensitivity across comparisons. The significant extension deficits at 0° and 15° showed moderate power (~0.63–0.72), supporting the reliability of these findings despite the modest sample size. Flexion at 0° also demonstrated an interpretable effect (power = 0.607). By contrast, non-significant comparisons (flexion at 10° and extension at 30°) showed very low power (~0.05–0.07), suggesting insufficient sensitivity rather than the absence of true group differences. Overall, the moderately powered results indicate a clinically meaningful imbalance in trunk strength, with reduced extensor capacity and relatively preserved or increased flexion torque in pain groups.

## 4. Discussion

The findings of the present study support and extend existing evidence that patients with low back pain (LBP) can benefit from the application of emerging technologies such as inertial measurement units (IMUs) in combination with electromyographic (EMG) analyses within a functional diagnostic setup [41,42].

Compared with MRI and CT imaging—which provide only static snapshots of the spine and its surrounding structures, including multifidus atrophy—IMU sensors enable the capture of kinematic data (e.g., acceleration and angular velocity) during real-life movements, while EMG signals simultaneously provide objective information on the electrical activation patterns of the musculature. This combined approach allows the diagnosis of deficits such as delayed anticipatory postural adjustments (APA) or a disturbed flexion–relaxation phenomenon.

Given the complex pathogenesis and diagnostic challenges of LBP—particularly with regard to the specification of treatment pathways and subsequent therapy stratification—a functional assessment should comprise analysis of gait phases, evaluation of neuromuscular control during stance, the assessment of movement control through flexion and extension bending tasks, the measurement of lumbopelvic stability via a modified slump test, and the determination of trunk muscle strength capacity. On the one hand, LBP patients frequently exhibit deficits in these subcategories compared with healthy individuals; on the other hand, such a comprehensive diagnostic approach allows for a highly differentiated characterization of neuromuscular deficits.

### 4.1. Functional Assessment

#### 4.1.1. Fatigue Resistance/Strength Endurance—Gait Analysis

Both the accelerometers positioned at the L3–L5 level and at the pelvis, as well as the EMG analysis of the bilateral multifidus (MF) muscles, revealed significant differences in the LBP group compared with the healthy control group. In the present study, IMU data from LBP patients demonstrated markedly altered movement dynamics, characterized by abrupt changes in acceleration with increasing pain intensity. This finding aligns with the results of [43] who examined gait phases in LBP patients during the remission phase. They attributed [43] the altered movement amplitudes to reduced MF activity during gait, resulting from increased fatty infiltration and neuromuscular dysfunction of the MF. This loss of stability leads to uncontrolled micro-movements of the spine at the lumbopelvic junction, further perpetuating the pain cycle [44].

Moreover, the duration of MF EMG activity was reduced in the LBP group. Similar findings were reported by [44] who investigated young asymptomatic individuals with a history of prolonged LBP episodes and likewise observed diminished EMG signals of the multifidus compared with healthy subjects. These changes suggest compensatory neuromuscular strategies that aim to stabilize the spine while simultaneously avoiding pain. This may be explained by reduced pelvic mobility due to increased pain-associated stiffness at the lumbopelvic junction [45,46].

However, these results stand in contrast to those of Bryndal et al., who demonstrated consistently higher MF muscle activation throughout the gait cycle in LBP patients compared with healthy participants [47]. The contradictory findings may be attributable to the tendency of LBP patients to exhibit inefficient neuromuscular activation of agonists and antagonists, even during gait. It is conceivable that baseline MF activation is initially higher than in healthy individuals but declines significantly with increasing walking duration and speed due to accelerated fatigue, resulting in a marked decrease in EMG signals over the course of the assessment [48,49]. Furthermore, both pain intensity and pain phase—acute versus chronic—may influence muscle activation and contribute to conflicting study outcomes [28].

#### 4.1.2. Neuromuscular Control

Consistent with previous studies, the pain groups exhibited increased center-of-pressure (CoP) track length and elevated power spectral density (PSD) during unilateral stance [50,51]. Additional significant differences were observed in the IMU sensor metrics as well as in EMG parameters of the multifidus (MF). The rise in spectral energy in both low- and high-frequency bands, together with altered power distribution along the X- and Y-axes, suggests axis-specific reweighting of postural control in LBP patients.

Despite the enlarged sway area, overall sway distance and velocity decreased, indicating reduced displacement amplitude but increased oscillatory frequency. Combined with the altered activation patterns of MF EMG parameters and the shifted minimum and maximum raw signal values of the erector spinae (ES), these findings point to a pain-related reorganization of trunk muscle activity during postural control, characterized by tonic changes in MF activation and greater involvement of the thoracic ES.

Both the significantly altered acceleration values and the modified sway patterns, along with changes in EMG parameters, may be explained by inconsistent activation and recruitment of the MF [52]. Overall, this pattern reflects a pain-related postural strategy marked by stiffer, higher-frequency control with broader spatial sway and redistribution of force across axes. Consequently, larger muscle groups such as the erector spinae assume movement control, leading to compensatory irregular acceleration patterns of the spine and sudden movements and braking behavior of the body’s center of mass during single-leg stance [50,53].

#### 4.1.3. Mobility Bending

Although the present study included lateral flexion and rotation bending tasks, significant differences between LBP patients and healthy participants were observed only in flexion and extension bending, both in IMU and EMG analyses. The pain group differed significantly in acceleration parameters as well as in MF EMG activity. Altered pelvic tilt combined with reduced MF EMG signals during flexion suggests increased tonic activation and diminished relaxation of the MF during the flexion phase. In contrast, MF EMG signals during extension were characterized by heightened tonic activation of thoracic and deep paraspinal muscles, likely reflecting protective co-contraction during re-extension.

Taken together, these findings indicate a pain-related movement strategy marked by restricted lumbar and pelvic motion and acceleration, accompanied by elevated baseline paraspinal activity consistent with a protective posture. IMU parameters revealed that LBP participants exhibited a reduced range of motion in both directions and lower overall acceleration throughout the bending trajectory. These deviations may be explained by movement-related fear (kinesiophobia) within the framework of the fear-avoidance model, as well as by insufficient extension strength [54,55].

Additionally, the LBP group demonstrated altered temporal coordination (timing) between movement (IMU) and muscle activation (EMG). While baseline MF EMG tension was elevated, maximum muscle activation was reduced compared with healthy controls [48,56]. On a neuro-muscular level, this may reflect inefficient MF activation combined with increased recruitment of the erector spinae [30]. Morphological fatty infiltration of the MF plays a critical role in these recruitment patterns and associated changes in motor control processes, progressively increasing from acute through subacute/recurring to chronic conditions [30].

#### 4.1.4. Lumbar–Pelvic Stability

In the present study, significant group differences were also observed during assessment of lumbopelvic stability using the sitting straight-leg-raise (SSLR) test. Analysis of IMU data indicated that the pain-free group was able to maintain stability of the lumbopelvic junction during leg elevation, whereas LBP patients exhibited an immediate posterior pelvic tilt. [57] attribute lateral pelvic tilt to the influence of insufficient lumbopelvic control [32,57].

The IMU data were temporally correlated with EMG recordings of the multifidus (MF). At the moment of pelvic tilting, EMG sensors showed a marked signal drop. Furthermore, MF EMG signals demonstrated a delayed onset, lagging behind the movement. Both factors suggest that movement control of the lumbopelvic junction is compensatory-shifted from the MF to the superficial back extensors (erector spinae). This less efficient neuromuscular control, combined with inadequate force transmission and increased instability of the lumbopelvic junction, may represent a risk factor for the development of LBP [28,58].

Although both low- and high-intensity pain groups exhibited significantly different values compared with the healthy group, causality in the high-intensity group is likely morphological, associated with advanced MF atrophy and fatty infiltration resulting from prolonged maladaptive loading. In contrast, the significantly altered values in the low-intensity group appear to be primarily attributable to deficits in neural activation [59].

#### 4.1.5. Core Strength

The isometric trunk strength assessment in flexion and extension revealed significantly reduced extension values alongside partially elevated flexion parameters in both pain groups compared with the healthy control group. Consequently, both pain groups deviate from the physiological norm and exhibit an impaired trunk strength balance between flexion and extension—a factor that must be considered a risk for the development and chronification of low back pain. Flexion dominance can be pathologically explained by a neuromuscular compensation strategy involving increased activation of the abdominal musculature (“bracing”) to stabilize the trunk, while extension (lordotic posture) is anticipated as painful. Simultaneously, inhibition of the extensors to avoid nociceptive stimuli may lead to reflexive suppression of deep spinal muscles, particularly the multifidus [32,59,60,61,62].

### 4.2. Limitations and Future Directions

Given the limited statistical power for some comparisons, the results should be interpreted with caution and confirmed in larger samples with appropriate multiplicity control. Measurement errors cannot be excluded, particularly due to repeated rotational and flexion movements at maximum range of motion. The close proximity of EMG and IMU sensors may have led to occasional contact during test execution, potentially causing artefacts that were erroneously interpreted as significant results. To minimize such misinterpretations, all initial statistical findings were independently reviewed for plausibility by two separate evaluators, and implausible values were excluded from the final analysis.

For improved accuracy, future test setups should include an additional IMU sensor at the thoracolumbar junction (T7–T1) to enable more precise assessment of overall spinal stability. Furthermore, clinical imaging of the spine was not available for the patients analyzed in this study; therefore, potential associations between inflammatory factors or other morphological pathologies and MF functional impairment could not be addressed and might be investigated in future research.

Future diagnostic procedures could increasingly rely on AI-based analysis methods and wearable systems to support continuous monitoring over longer periods in everyday life. Such an approach could help reduce dependence on artificially modeled laboratory scenarios and instead depict the development of functional deficits in a more life-like context.

In order to differentiate more precisely between pathological movement patterns and adaptive compensation in the future, the fear avoidance beliefs questionnaire should be used, as these often correlate with pathological movement patterns, while adaptive compensations are more frequently found in patients with high functional independence in everyday life.

Furthermore, when considering the results, it must be taken into account that the requirement to stand on one leg for 60 s may bias the sample towards more capable individuals.

## 5. Conclusions

Nevertheless, the integrated application of novel technological devices in combination with functional screening already offers the opportunity to shift LBP diagnostics from a primarily imaging-based approach to a functional paradigm. Incorporating objective measurement data enabled by wearables and technical devices can lead to a deeper understanding of disease mechanisms and pain genesis, fundamentally transforming clinical evaluation and also therapeutic strategies.

This is particularly relevant in light of findings by M. O’Keeffe et al., who demonstrated that accurate diagnostic assessment of back pain has therapeutic potential: specifically identifying the causal pain mechanism reduces the need for surgical interventions or further imaging and simultaneously improves both subjective pain experience and patient prognosis [63,64,65].

Although MRI and CT remain indispensable for identifying pathologies as fractures or tumors (among other “red flags”), the combination of EMG and IMU represents a superior tool for diagnosing the mechanical and neuromuscular causes of chronic back pain.

The combination of these methods permits conclusions about underlying functional relationships: While IMUs provide indications of mechanical abnormalities (e.g., stiffness or instability), EMG points to potential neuromuscular factors—such as altered activation of the multifidus muscle. This supports differentiated therapy planning that goes beyond subjective pain scales and standardized training programs.

The diagnostic tool set is radiation-free, cost-effective, and reliable. Furthermore, these technologies allow the objective re-evaluation of neuromuscular adaptation progress (e.g., following stabilization training) and provide patients with direct feedback on muscle activation through biofeedback.

In routine clinical settings, an IMU-based sensor setup is recommended. Its straightforward operation facilitates time-efficient placement on the patient (“plug-and-play”). The advantage of this diagnostic method lies in real-time data generation during functional load tests. This type of data acquisition enables functional phenotyping, allowing patients to be stratified into functional subgroups and treatment courses to be tailored accordingly.

Basic research in laboratory settings should utilize a combined EMG-IMU setup for further diagnostic evaluation. While this is very complex and time-consuming in its practical application, it can provide significantly more specific insights into functional compensation patterns for the scientific validation of therapies or the elucidation of causal relationships between pain and functional limitations.

## Figures and Tables

**Table 1:** Anthropometric data of the study sample.

Group	Male [*n*]	Height [cm]	Weight [kg]	Age [years]
No pain	2	1760 (2.8)	82.5 (13.4)	34.0 (7.1)
Low-intensity pain	6	181.3 (5.9)	89.6 (19.8)	40.1 (8.3)
High-intensity pain	5	182.8 (7.8)	87.0 (13.2)	40.2 (7.2)
Group	Female [*n*]	Height [cm]	Weight [kg]	Age [years]
No pain	7	170.5 (5.2)	66.9 (8.0)	44.7 (11.1)
Low-intensity pain	8	169.6 (6.8)	69.2 (11.6)	46.8 (16.1)
High-intensity pain	10	167.4 (4.1)	70.06 (13.2)	45.7 (10.4)

**Table 2:** Functional assessment screening protocol.

Functional Level	Assessment	Technical Equipment
Fatigue resistance/strength endurance	100 m gait test;	EMG and IMU
Mobility and neuromuscular control (CoP)	60 s bipedal and monopedal (left and right) stance with eyes open; Spinal ROM tests while standing and sitting, every movement twice: flexion/extension, lateral bending and rotation; 60 s upright sitting with eyes open	CoP-force plate EMG and IMU
Lumbopelvic stability	Modified slump test	EMG and IMU
Global trunk musculature	times for 6 s isometric trunk strength measurements in the sagittal plane in different flexion angular positions of the hip: 90°, 80°, 105° and 120°	IsoMed 2000 (D. & R. Ferstl GmbH, Hemau, Germany)

**Table 3:** Overview of the significant measurement results across the three pain groups in the gait analysis.

Position	Measurement	*p*-Value	Power	Mean No Pain	Mean Low-Intensity Pain	Mean High-Intensity Pain
Pelvis_Acc_X	max_slopeR	0.043	0.558	0.02	0.02	0.035
R_Multifidii	max_slopeL	0.049	0.488	0.02	0.03	0.03
Pelvis_Acc_X	max_steigungR	0.043	0.558	0.02	0.02	0.035
Left_Thigh_Acc_Z	varL	0.042	0.562	0.03	0.04	0.063
Left_Thigh_Acc_Z	max_slopeR	0.012	0.759	0.01	0.02	0.028
Left_Thigh_Acc_Z	max_steigungR	0.012	0.759	0.01	0.02	0.02
L_Multifidii	meanR	0.05	0.505	0.0009	0.0007	0.0005
R_Multifidii	aucR	0.046	0.509	0.07	0.07	0.05

**Table 4:** Overview of the significant measurement results across the three pain groups in the CoP analysis.

Measurement	*p*-Value	Power	Mean No Pain	Mean Low-Intensity Pain	Mean High-Intensity Pain
FreqBandEnergy_L	0.023	0.661	2.54 × 10^14^	2.7 × 10^14^	3.98 × 10^14^
AreaTrembling	0.033	0.599	1.53 × 10^14^	4.46 × 10^14^	4.2 × 10^14^
SwayArea_Ellipse	0.024	0.654	1.99 × 10^14^	4.3 × 10^14^	4.61 × 10^14^
MeanFreq_X	0.048	0.505	2.30 × 10^14^	3.34 × 10^14^	3.82 × 10^14^
Power_Y	0.031	0.608	1.99 × 10^14^	2.3 × 10^14^	2.67 × 10^14^
Power_X	0.027	0.634	3.6 × 10^14^	2.94 × 10^14^	1.72 × 10^14^
DistanceTrembling	0.05	0.523	5.0 × 10^14^	4.58 × 10^14^	3.87 × 10^14^
FreqBandEnergy_H	0.019	0.691	3.06 × 10^14^	3.89 × 10^14^	5.72 × 10^14^
VelocityTrembling	0.04	0.57	3.88 × 10^14^	3.07 × 10^14^	1.65 × 10^14^
DominantFreq_Y	0.049	0.497	152,587,890.6	3.33 × 10^8^	6.1 × 10^8^

**Table 5:** EMG of trunk muscles during single-leg stance across pain conditions: minimum envelope and raw values (means, uncorrected *p* values, and post hoc power).

Position	Measurement	*p*-Value	Power	Mean No Pain	Mean Low-Intensity Pain	Mean High-Intensity Pain
R_Int_Oblique	minENV	0.033	0.60	−7.48	−0.05	0.54
L_Multifidii	minENV	0.001	0.93	2.12	0.81	−0.47
R_ThoracicEs	minRAW	0.055	0.47	−81.22	−102.67	−166.51
R_ThoracicEs	maxRAW	0.052	0.48	76.29	109.66	181.36
L_ThoracicEs	minRAW	0.047	0.53	−96.84	−83.80	−163.481
R_Multifidii	minENV	0.012	0.75	−2.07	−0.45	1.60

**Table 6:** Evaluation of bending movement in a seated position: The upper body is bent forward from an upright position into extension.

Measurement	*p*-Value	Power	Mean No Pain	Mean Low-Intensity Pain	Mean High-Intensity Pain
LWS_Acc_X_max	0.024	0.658	1.185	0.92	0.84
LWS_Acc_X_range	0.03	0.623	1.18	0.94	0.85
LWS_roll_max	0.019	0.69	78.44	68.40	60.99
LWS_roll_range	0.023	0.665	81.93	65.67	58.26
Pelvis_Acc_X_max	0.03	0.62	0.940	0.68	0.64
Pelvis_Acc_X_range	0.03	0.623	0.93	0.68	0.64
Pelvis_roll_max	0.028	0.632	58.19	47.66	42.95
R_Multifidii_env_min	0.049	0.512	3.14	3.20	6.66
L_Multifidii_env_min	0.034	0.598	2.79	3.38	7.99
LWS_Acc_X_area	0.037	0.582	3.02	2.34	1.92
lws_pelvis_pitch_min	0.003	0.905	4.47	0.43	0.06

**Table 7:** Evaluation of bending in a seated position: The upper body is raised from the flexion position into extension.

Measurement	*p*-Value	Power	Mean No Pain	Mean Low-Intensity Pain	Mean High-Intensity Pain
LWS_Acc_X_area	0.012	0.753	4.03	2.66	2.37
lws_pelvis_pitch_min	0.009	0.798	6.5	1.99	0.09
R_ThoracicEs_env_mean	0.035	0.594	13.46	18.02	23.08
R_ThoracicEs_env_RMS	0.039	0.576	15.51	21.55	26.43
L_Multifidii_DutyCycle	0.042	0.563	0.71	0.77	0.88
LWS_Acc_X_area	0.005	0.845	3.90	2.82	2.24
LWS_Acc_Y_min	0.029	0.626	−0.12	−0.06	−0.041
lws_pelvis_pitch_area	0.021	0.681	216.87	123.89	125.03
LWS_pitch_area	0.031	0.613	426.20	342.91	286.77
LWS_roll_range	0.031	0.617	102.96	65.04	62.29
Pelvis_Acc_X_area	0.017	0.711	2.91	2.18	1.68
pelvis_thigh_roll_area	0.018	0.705	175.35	125.86	117.24
R_Multifidii_env_min	0.016	0.719	2.33	2.48	6.02
R_ThoracicEs_raw_mean	0.036	0.587	0.00	0.007	0.016

**Table 8:** Electromyographic trunk muscle responses during the seated straight leg raise across pain groups.

Measurement	Term	*p*-Value	Power	Mean No Pain	Mean Low-Intensity Pain	Mean High-Intensity Pain
L_Multifidii	minENV	0.033	0.6	1.8	0.43	−0.83
R_Int_Oblique	meanRAW	0.053	0.43	−0.00018	0.0004	0.000721
L_ThoracicEs	meanRAW	0.050	0.41	−0.00049	3.94 × 10^−5^	0.000743

**Table 9:** Isometric trunk strength normalized to body mass across pain conditions.

Measurement	*p*-Value	Power	Mean No Pain Nm/kg	Mean Low-Intensity Pain Nm/kg	Mean High-Intensity Pain Nm/kg
flex_max_0	0.033	0.607	1.28	1.31	1.67
flex_max_10	0.702	0.067	1.24	1.22	1.58
ext_max_0	0.029	0.634	3.01	2.68	2.62
ext_max_15	0.016	0.724	3.19	2.78	2.73
ext_max_30	0.879	0.053	3.13	2.71	2.82

## Data Availability

The original contributions presented in this study are included in the article. Further inquiries can be directed to the corresponding author.

## Appendix A

### Appendix A.1. Search Strings

#### Search Strategy

Population: LBP

Intervention: clinical/functional assessments and EMG

Outcome: functional screening

Comparison: n.a.

Setting: RCT, SR, CT

	**Identification**	
**Database**	**Search Query**	**Hits**
PubMed	((((“Low Back Pain” OR “LBP”) AND (“functional movement screen*” OR “functional assessment” OR “assessment instruments” OR assessment* OR FMS OR “EMG” OR “NRS”)) AND (diagn* OR prevent* OR control)) NOT (therapy OR reha* OR fractur* OR pregnancy OR imag* OR prevalence OR EEG OR effect* OR stress OR question* OR biomark* OR inflam*))	*n* = 296
CINAHL		*n* = 40
	Screening	
Results reviewed for duplicates		*n* = 312
Excluded after title abstract screening		*n* = 213
Full texts checked for suitability		*n* = 99
	Full texts checked for suitability	*n* = 99
Excluded	Reason	Case number
	No access to full text/no full text (too old)	*n* = 29
Population	Different search terms (no LBP)	*n* = 35
Intervention	Different intervention	*n* = 12
Outcome	Different outcome	*n* = 13
Studies included in review		*n* = 10

## Appendix B

### Appendix B.1. Detailed Description of IMU-Based Kinematic Features Gait Analysis

**Muscle/Sensor**	**Metric**	**Meaning**	**Calculation**	**Interpretation**
Pelvis_Acc_X	max_slopeR	Maximum change (slope) of the acceleration signal between two consecutive samples	Difference of the time series × sampling frequency (first time derivative)	High values indicate abrupt, jerky pelvic movements → suggestive of instability or pain-related protective reactions
R_Multifidii (EMG)	max_slopeL	Maximum rate of change of the EMG envelope per unit time	Difference of the EMG envelope × fs	Reflects the speed of changes in muscle activation; high values = rapid recruitment increases
Pelvis_Acc_X	max_steigungR	Maximum change (slope) of the acceleration signal between two consecutive samples	Same as above	Same as above
Left_Thigh_Acc_Z	varL	Variance of the Z-axis signal	Variance of the time series	Higher variance = more irregular, less controlled thigh movement
Left_Thigh_Acc_Z	max_slopeR	Maximum slope of the Z-axis acceleration signal	First derivative (diff × fs)	High peaks = abrupt vertical acceleration changes during gait or bending
Left_Thigh_Acc_Z	max_steigungR	Identical to max_slopeR	Same as above	Same as above
L_Multifidii (EMG)	meanR	Mean value of the EMG envelope over the analysis window	Averaging the EMG envelope	Elevated values = increased tonic activation; reduced values may indicate inhibition or fatigue
R_Multifidii (EMG)	aucR	Area under the EMG envelope (integral activation)	Trapezoidal integration of the EMG envelope	High AUC = greater overall activation load; low AUC = reduced recruitment or inhibition

### Appendix B.2. Detailed Overview of CoP, Frequency-Band Energy, and Trembling-Related Metrics

**Measurement**	**Meaning**	**Calculation (Description)**	**Interpretation**
FreqBandEnergy_L	Energy in the low-frequency band (<0.3 Hz)	Sum of spectral magnitudes in the low-frequency band (freq < 0.3 Hz)	Higher values indicate a greater contribution of slow sway components, typically reflecting reduced postural stability due to larger, slower corrective movements
AreaTrembling	Area under the trembling curve (high-frequency CoP component)	Numerical integration of the trembling component using the trapezoidal rule	Larger area reflects stronger micro-sway, indicating reduced postural control and less efficient neuromuscular regulation
SwayArea_Ellipse	Ellipse covering the CoP sway (95% confidence ellipse)	Calculation of the 95% confidence ellipse around the CoP trajectory	Larger ellipse area indicates greater sway amplitude, suggestive of poorer overall postural stability
MeanFreq_X	Weighted mean frequency of the CoP signal in X-direction	mean_freq_x = Σ(f × Pxx)/Σ(Pxx)	Higher values indicate stronger involvement of higher frequencies → stiffer, faster corrective adjustments by the neuromuscular system
Power_Y	Maximum spectral power contribution in the Y-direction	Dominant peak in the Y-axis power spectrum	Higher values reflect dominant lateral sway activity, potentially associated with compensatory strategies or side-specific instability
Power_X	Maximum spectral power contribution in the X-direction	Dominant peak in the X-axis power spectrum	Lower values indicate reduced anterior–posterior sway, often associated with pain-related stiffness strategies
DistanceTrembling	Total path length of trembling (micro-sway trajectory)	Sum of Euclidean distances between consecutive trembling points	Higher distance indicates increased neuromuscular effort, often inefficient or compensatory in individuals with pain
FreqBandEnergy_H	Energy in the high-frequency band (1–3 Hz)	Sum of spectral magnitudes in the high-frequency band (1 ≤ freq ≤ 3 Hz)	Higher values indicate more high-frequency compensatory movements, reflecting stiffer, faster but less efficient postural control
VelocityTrembling	Mean trembling velocity	Mean Euclidean point-to-point velocity × sampling frequency	Higher values indicate accelerated micro-corrections, suggesting over-compensation or instability
DominantFreq_Y	Frequency of the highest spectral peak in Y-direction	Frequency location of the dominant peak in the Y-axis signal	Higher values indicate dominant high-frequency lateral corrections, typical in conditions of pain, stiffness, or compensatory behavior

### Appendix B.3. EMG-Based Trunk Muscle Parameters: Definitions, Computation, and Interpretation During CoP Measurement

**Muscle/Sensor**	**Metric**	**Meaning**	**Calculation**	**Interpretation**
R_Int_Oblique	minENV	Minimum value of the EMG envelope	min(env)	High (relative): elevated baseline activation, potential excessive muscle tension. Low: adequate relaxation phases, energy-efficient control.
L_Multifidii	minENV	Minimum value of the EMG envelope	min(env)	High (relative): increased tonic activity, possible protective overactivation. Low: sufficient relaxation, physiologically efficient.
R_ThoracicEs	minRAW	Minimum value of the raw EMG signal	min(raw)	Very low values may indicate artefacts; moderately low values fall within a plausible physiological range.
R_ThoracicEs	maxRAW	Maximum value of the raw EMG signal	max(raw)	High peaks: strong activation spikes, possibly reflecting neuromuscular compensation or signal distortion. Low values: typical physiological activity.
L_ThoracicEs	minRAW	Minimum value of the raw EMG signal	min(raw)	Extremely low values may reflect noise or artefacts; moderately low values are physiologically plausible.
R_Multifidii	minENV	Minimum value of the EMG envelope	min(env)	High (relative): increased tonic baseline activation, potential muscle guarding. Low: appropriate relaxation capability and efficient neuromuscular control.

### Appendix B.4. Definitions and Interpretation of IMU and EMG Features Extracted During Seated Flexion and Extension Movements

**Muscle/Sensor**	**Metric**	**Meaning**	**Calculation**	**Interpretation**
LWS	Acc_X_max	Maximum lumbar acceleration in X-direction	max(LWS_Acc_X)	High values: abrupt movements or compensatory evasive strategies; low values: controlled or reduced lumbar motion
LWS	Acc_X_range	Acceleration range (max–min) of the lumbar signal	max(LWS_Acc_X)–min(LWS_Acc_X)	High: large movement amplitude, potential overload; low: restricted mobility
LWS	roll_max	Maximum roll angle of the lumbar spine	Maximum of the roll signal	High: strong lateral motion; low: restricted or pain-related “stiffness”
LWS	roll_range	Roll angle range	max–min of the roll angle	High: large lateral mobility; low: stabilized or pain-limited movement
Pelvis	Acc_X_max	Maximum pelvic acceleration in X-direction	Maximum of pelvic_acc_x	High peaks: jerky pelvic motion, instability
Pelvis	Acc_X_range	Acceleration range of the pelvis (X)	max–min	Large: high mobility; small: restricted pelvic tilt
Pelvis	roll_max	Maximum roll angle of the pelvis	Maximum of pelvic roll	Larger values indicate lateral compensation; high = reduced stability
R. Multifidus (EMG)	env_min	Minimum value of the EMG envelope (right side)	min(envelope)	High: minimal relaxation, tonic overactivation; low: normal relaxation
L. Multifidus (EMG)	env_min	Minimum value of the EMG envelope (left side)	min(envelope)	High: compensatory baseline tension; low: good relaxation capability
LWS	area	Integrated lumbar acceleration over time	Area under the curve (trapezoidal integration)	High: large overall movement; low: restricted mobility
LWS/Pelvis	pitch_min	Minimum pitch angle between lumbar spine and pelvis	min(θ(t))	Near zero: strongly reduced flexion → pain or guarding; more negative values = full flexion
Thoracic ES (Right)	env_mean	Mean EMG envelope of the thoracic erector spinae	mean(envelope)	High: compensatory activation/load transfer; low: efficient posture control
Thoracic ES (Right)	env_RMS	RMS of the EMG envelope (energy-related activation measure)	RMS(envelope)	High: elevated muscular energy → fatigue risk
L. Multifidus (EMG)	DutyCycle	Proportion of time the MF is active	Share of samples > 20% of maximal envelope	High: static load, less relaxation; low: efficient intermittent activation
LWS	Acc_Y_min	Minimum lumbar acceleration in Y-direction	min(Acc_Y)	Low values: downward/backward motion → possible pain-avoidance behavior
LWS	Acc_Y_area	Integrated pitch-related lumbar motion	Area under the pitch curve	Low: reduced lumbar/pelvic motion, increased stiffness
Pelvis	pelvis_pitch_area	Total pitch motion between lumbar spine and pelvis	Trapezoidal integration	Large: high movement range; low: pain-related movement restriction
LWS	pitch_area	Integrated lumbar pitch motion	Trapezoidal integration	High: greater movement amplitude; low: restricted motion
LWS	roll_range	Roll movement range of the lumbar spine	max–min of roll	High: potential instability; low: restricted mobility
Pelvis	Acc_X_area	Integrated pelvic acceleration in X-direction	Area under the curve	High: dynamic pelvic behavior; low: stiff/restricted pelvis
Pelvis	thigh_roll_area	Integrated roll movement between pelvis and thigh	Trapezoidal integration	High: increased movement magnitude; low: restricted movement
R. Multifidus (EMG)	env_min	Minimum EMG envelope (again)	min(envelope)	High: tonic overactivation; low: good relaxation ability
Thoracic ES (Right)	raw_mean	Mean raw EMG value	mean(raw EMG)	High: increased muscle activity; low: efficient baseline activation
